# Comparison of the clinical manifestations and chest CT findings of pulmonary cryptococcosis in immunocompetent and immunocompromised patients: a systematic review and meta-analysis

**DOI:** 10.1186/s12890-022-02175-9

**Published:** 2022-11-11

**Authors:** Chunlin Xiong, Jianguo Lu, Ting Chen, Rui Xu

**Affiliations:** 1 Department of Radiology, The Second Affiliated Hospital of Chongqing Medical University, Chongqing, 400010 People’s Republic of China; 2 Department of Cardiology, People’s Hospital of Fengjie, Fengjie, Chongqing, 404600 People’s Republic of China; 3 Department of Radiology, People’s Hospital of Fengjie, Fengjie, Chongqing, 404600 People’s Republic of China; 4 Department of Respiratory Medicine, The Second Affiliated Hospital of Chongqing Medical University, Chongqing, 400010 People’s Republic of China

**Keywords:** Meta-analysis, Clinical manifestations, Radiologic findings, Pulmonary cryptococcosis, Immunocompetent, Immunocompromised

## Abstract

**Objective:**

The purpose of our study was to perform a meta-analysis and systematic review to compare differences in clinical manifestations and chest computed tomography (CT) findings between immunocompetent and immunocompromised pulmonary cryptococcosis (PC) patients.

**Methods:**

An extensive search for relevant studies was performed using the PubMed, EMBASE, Cochrane Library, and Web of Sciences databases from inception to September 30, 2021. We included studies that compared the clinical manifestations and chest CT findings between immunocompetent and immunocompromised PC patients. Study bias and quality assessment were performed using the Newcastle–Ottawa Scale (NOS).

**Results:**

Nine studies involving 248 immunocompromised and 276 immunocompetent PC patients were included in our analysis. The NOS score of each eligible study was above 5, indicating moderate bias. The proportion of elderly patients (> = 60 years old) in the immunosuppressed group was significantly higher than that in the immunocompetent group (OR = 2.90, 95% CI (1.31–6.43), Z = 2.63, *p* = 0.01). Fever (OR = 7.10, 95% CI (3.84–13.12), Z = 6.25, *p* < 0.000) and headache (OR = 6.92, 95% CI (2.95–16.26), Z = 4.44, p < 0.000) were more common in immunosuppressed patients. According to thin-section CT findings, lesions were more frequently distributed in the upper lobe (OR = 1.90, 95% CI (1.07–3.37), Z = 2.2, *p* = 0.028) in immunocompromised individuals. The proportions of patients with cavity sign (OR = 5.11, 95% CI (2.96–8.83), Z = 5.86, *p* = 0.00), ground-glass attenuation (OR = 5.27, 95% CI (1.60–17.35), Z = 2.73, *p* = 0.01), and mediastinal lymph node enlargement (OR = 2.41, 95% CI (1.12–5.20), Z = 2.24, *p* = 0.03) were significantly higher in immunocompromised patients.

**Conclusion:**

No significant differences in nonspecific respiratory symptoms were found between immunocompromised and immunocompetent PC patients. Nevertheless, fever and headache were more common in immunocompromised patients. Among the CT findings, cavity, ground-glass attenuation, and mediastinal lymph node enlargement were more common in immunocompromised individuals.

**Supplementary Information:**

The online version contains supplementary material available at 10.1186/s12890-022-02175-9.

## Background

Pulmonary cryptococcosis (PC) is an invasive pulmonary mycosis caused by pathogenic *cryptococcus* infection. The major pathogenic *cryptococcus* species that cause PC are *Cryptococcus neoformans* and *Cryptococcus gattii* [1, 2]. The major route of PC infection is inhalation of cryptococcal spores from aerosols. Due to the clinical application of immunosuppressants, checkpoint inhibitors, chemotherapeutics, glucocorticoids and other drugs that suppress the immune system, the incidence of PC has increased rapidly in recent years [3]. PC has a high affinity for the central nervous system in immunocompromised patients, potentially resulting in cryptococcal meningoencephalitis. Globally, approximately 1 million immunocompromised patients develop cryptococcal meningitis each year, with a 60% mortality rate at 3 months after central nervous system infection [4]. In USA, the incident rate is 0.4–1.3/1,000,000. The incident rate of PC rises to 2–7/1,000,000 in patients with HIV and AIDS [5]. The pathogenesis of PC is related to defects in immune function. Thus, immunocompromised hosts are more susceptible to PC. However, PC can also occur in immunocompetent subjects. With the development of new diagnostic techniques, the rate of PC detection in immunocompetent patients has increased in recent years [6, 7].

Patients with localized PC often present with nonspecific respiratory or systemic symptoms, such as cough, expectoration, dyspnea, chest pain, and fever. Different from other fungal infections, such as typical Pulmonary Aspergillus Overlap Syndromes (PAOS) caused by invasive aspergillosis [8], some patients may be asymptomatic [9]. Compared with chronic aspergillus infection with typical air-crescent sign, PC lacks typical CT manifestations [10]. PC characterized by single or multiple nodules is more easily misdiagnosed as peripheral lung cancer or tuberculosis. Some studies focused on the CT manifestations of PC in immunocompromised or immunocompetent patients and identified some thin-section CT features, such as halo signs, solitary or multiple nodules, and so on [11]. However, these radiologic features are nonspecific. Due to nonspecific clinical manifestations and radiologic features, PC patients are often misdiagnosed with bacterial or organizing pneumonia, tuberculosis, or even lung cancer at their initial visit.

Due to the different immune responses elicited by Cryptococcus infection in immunocompromised and immunocompetent patients, immunocompetent and immunosuppressed PC patients exhibit some different clinical manifestations and radiologic findings. Thus, several studies have focused on comparisons of the clinical features and radiologic findings between immunocompetent and immunosuppressed PC patients. Although several reviews have summarized the possible clinical manifestations and imaging features of PC, few meta-analyses have confirmed the different clinical features and radiologic findings between immunocompetent and immunosuppressed patients [12, 13]. In this systematic review and meta-analysis, we aimed to comparing clinical characteristics and radiologic features between immunocompetent and immunosuppressed patients.

## Methods

### Search strategy and eligibility criteria

Our systematic review was in accordance with the Preferred Reporting Items for Systematic Reviews and Meta-Analyses (PRISMA) statement [14]. An extensive search for relevant studies was performed using the PubMed, EMBASE, Cochrane Library, and Web of Sciences databases from inception to September 30, 2021. The search keywords and the related syntax were (“pulmonary cryptococcosis” OR “lung criptococcosis”) AND “immunocompetent” AND “immunocompromised”. We also checked the references of key articles for any additional eligible articles. Studies were selected if they met the following eligibility criteria: (1) chest CT was used in the diagnosis of PC, and (2) the diagnosis of PC was based on percutaneous biopsy, surgical resection, bronchoalveolar lavage, transbronchial biopsy or culture. We excluded duplicate reports, editorials, correspondences, conference abstracts, commentaries and case reports. The selection of suitable articles was performed by 2 investigators independently. Disparities between investigators were resolved by consensus of all the investigators.

### Data extraction and quality assessment

Two independent researchers (C. X and J. L) performed data extraction and evaluated the literature quality. Disagreements were resolved by consensus of all the investigators. We extracted the following variables from each included study: first author, publishing institution, publication time, number of immunocompromised or immunocompetent patients with PC, patient sex, patient age, clinical symptoms, lesion distributions, and radiologic features. Study bias and quality assessment were performed independently by two authors (T.C and R. X) using the Newcastle–Ottawa Scale (NOS). The NOS consists of 3 sections, selection, comparability, and exposure, with a maximum score of 9 points. Total scores of 0–3 indicate poor quality, scores of 4–6 indicate fair quality, and scores of 7–9 represent high quality [15]. Discrepancies were resolved by discussion with all investigators. The scores of the included studies are shown in Additional file 1: Table S1.

### Statistical analysis

We used STATA SE version 15.1 software and a random-effects model to calculate pooled prevalence rates (elderly patient ratio, patient sex, clinical symptoms, and CT characteristics) with corresponding 95% confidence intervals (CIs) for clinical data. Pooled odds ratios (ORs) with 95% CIs for the elderly patient ratio, patient sex, clinical symptoms, and CT characteristics in immunocompetent and immunocompromised patients were calculated with STATA SE version 15.1 software. Heterogeneity among the included studies was assessed using Cochran’s *Q* test and the *I*^2^ statistic. When *I*^2^ < 50%, a fixed-effect model was chosen; otherwise, a random-effects model was selected. *p* < 0.05 was considered to be statistically significant. Publication bias was evaluated by Begg’s test and Egger’s test.

## Results

### Characteristics of the studies and quality assessments

**Fig. 1:**
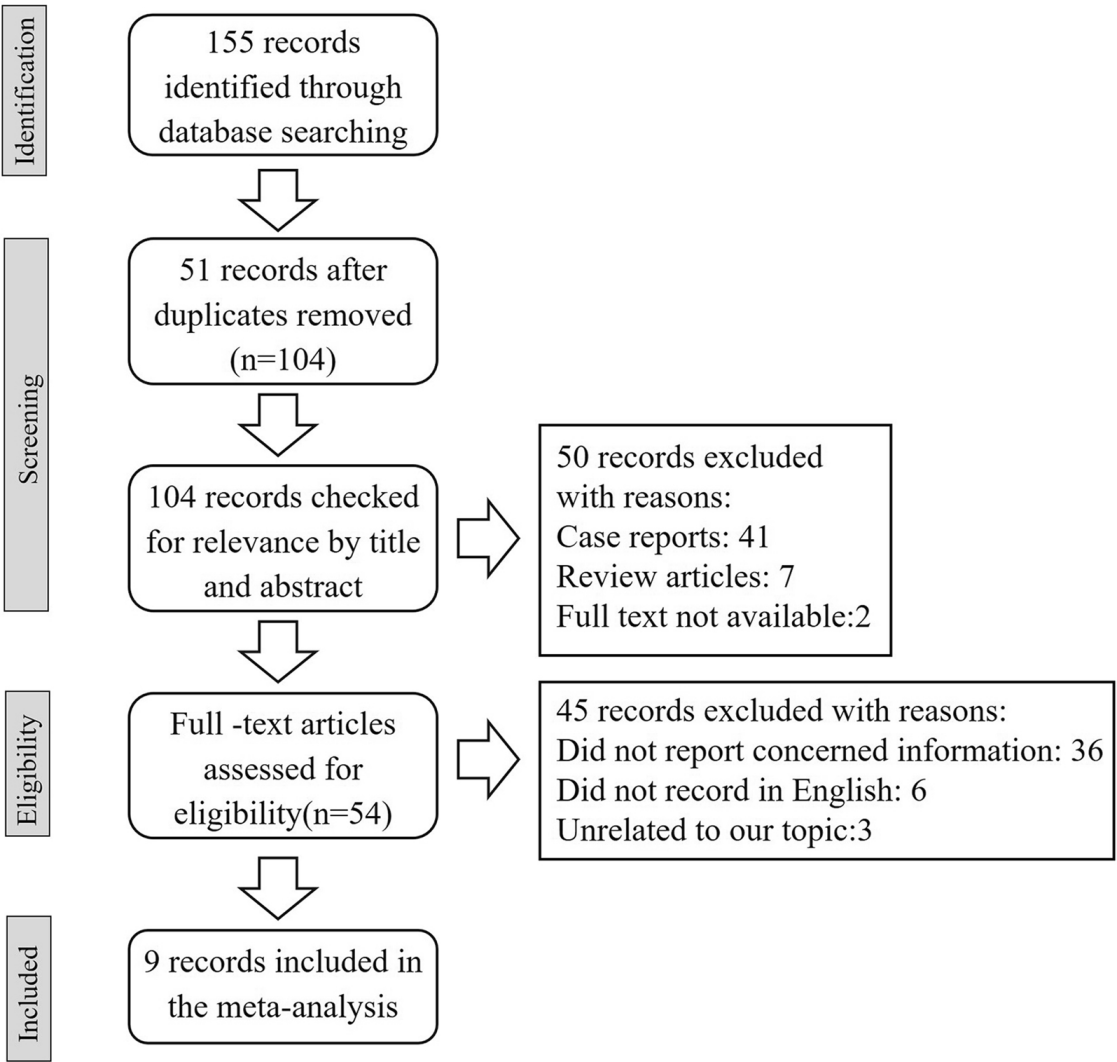
Flow diagram of the study selection process

The study selection process is shown in Fig. 1. In brief, 155 references were collected after searching the databases. Fifty-one references were removed due to duplication. After scanning the titles and abstracts, 95 records were excluded for the reasons listed in Fig. 1. Finally, 9 full texts, including 248 immunocompromised and 276 immunocompetent patients, were assessed for eligibility and included in our meta-analysis [16–24].

### Demographic characteristics of the patients with PC in the immunocompetent and immunocompromised groups

**Fig. 2:**
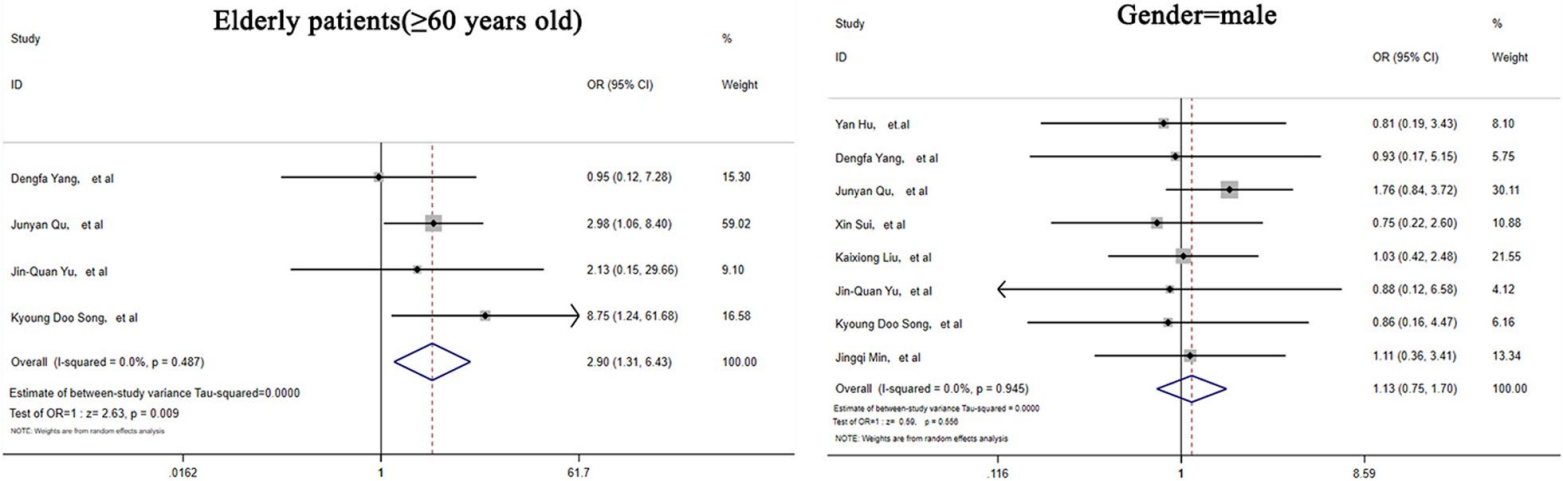
Forest plots depicting the comparisons of demographic characteristics in immunocompetent and immunocompromised PC patients

The demographic characteristics of the patients included in the relevant studies are listed in Table 1. The causes for immunosuppressed status in the included studies were broadly classified into AIDS, organ transplantation, diabetes mellitus, immunosuppressive drugs or corticosteroids used for basic illness, hematological malignancies, solid tumor, and connective tissue disorders. Of the patients recorded in the selected articles, 33.0% (95% CI: 16.7–49.4, *I*^2^ = 50.5%) of immunocompromised patients were elderly patients (> = 60 years old), while only 13.2% (95% CI: 6.1–20.3, *I*^2^ = 0.0%) of immunocompetent patients were elderly patients (> = 60 years old). The proportion of elderly patients in the immunosuppressed group was significantly higher than that in the immunocompetent group (OR = 2.90, 95% CI (1.31–6.43), Z = 2.63, *p* = 0.01). Among these patients, 62.8% (95% CI: 56.2–69.3, *I*^2^ = 0.0%) were immunosuppressed, and 59.2% (95% CI: 53.1–65.3, *I*^2^ = 0.0%) were male. There were no significant differences between the two immune status groups with respect to sex (OR = 1.13, 95% CI (0.75–1.70), Z = 0.59, *p* = 0.56) (Fig. 2).

**Table 1:** Demographic characteristics of the immunocompetent and immunocompromised patients with PC

Study	Immunocompromised patients			Immunocompetent patients		
	Total patients	Age > = 60	Male	Total patients	Age > = 60	Male
Yan Hu, et al	10	NA	5	29	NA	16
Dengfa Yang, et al	9	2	4	13	3	6
Junyan Qu, et al	94	27	64	42	5	23
Xin Sui, et al	24	NA	13	18	NA	11
Kaixiong Liu, et al	35	NA	22	53	NA	33
Lixuan Xie, et al	43	NA	NA	29	NA	NA
Jinquan Yu, et al	5	1	3	19	2	12
Kyoung Doo Song, et al	11	7	6	12	2	7
Jingqi Min, et al	17	NA	11	61	NA	38

### Clinical features of the patients with PC in the immunocompetent and immunocompromised groups

**Fig. 3:**
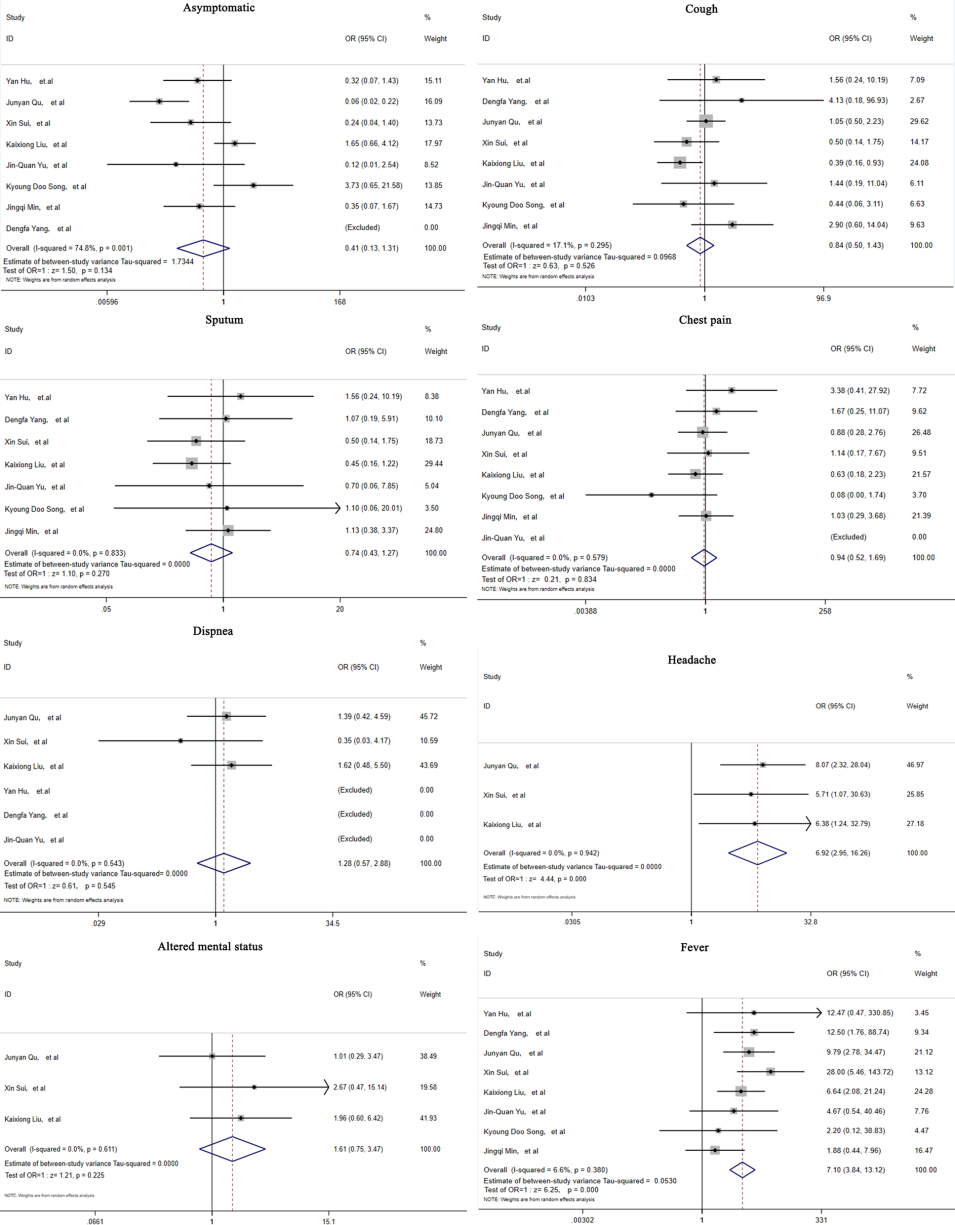
Forest plots depicting the comparisons of clinical features in immunocompetent and immunocompromised PC patients

The general clinical features of PC in the immunocompetent and immunocompromised groups are listed in Table 2. Among all the PC patients, 40.8% immunocompetent and 30.2% immunocompromised patients were asymptomatic. However, no statistic differences were found. Regarding clinical manifestations in immunocompromised patients, cough was reported in 43.6% (95% CI: 20.0–67.3, *I*^2^ = 77.5%), expectoration was reported in 35.5% (95% CI: 20.6–50.5, *I*^2^ = 0.0%), chest pain was reported in 12.6% (95% CI: 7.9–17.3, *I*^2^ = 0.0%), fever was reported in 26.5% (95% CI: 14.9–38.0, *I*^2^ = 70.3%), dyspnea was reported in 10.6% (95% CI: 3.6–17.6, *I*^2^ = 49.1%), and headache was reported in 32.8% (95% CI: 19.6–46.0, *I*^2^ = 64.0%). A total of 15.7% (95% CI: 6.1–25.4, *I*^2^ = 51.9%) of immunocompromised PC patients had altered mental status. Among the immunocompetent patients, cough was reported in 52.3% (95% CI: 36.6–68.0, *I*^2^ = 69.1%), expectoration was reported in 40.2% (95% CI: 27.0–53.4, *I*^2^ = 27.9%), chest pain was reported in 14.7% (95% CI: 10.2–19.2, *I*^2^ = 27.1%), fever was reported in 5.1% (95% CI: 2.4–7.7, *I*^2^ = 26.3%), dyspnea was reported in 10.6%(95% CI: 4.9–16.2, *I*^2^ = 0.0%), and headache was reported in 5.3% (95% CI: 1.2–9.4, *I*^2^ = 0.0%). A total of 10.6% (95% CI: 4.9–16.2, *I*^2^ = 0.0%) of immunocompromised PC patients had altered mental status. Furthermore, we investigated the differences in clinical manifestations between immunocompromised and immunocompetent PC patients. Among all the clinical symptoms, fever (OR = 7.10, 95% CI (3.84–13.12), Z = 6.25, *p* < 0.000) and headache (OR = 6.92, 95% CI (2.95–16.26), Z = 4.44, p < 0.000) were significantly more prevalent in immunocompromised PC patients (Fig. 3).

**Table 2:** Clinical manifestations in the immunocompetent and immunocompromised patients with PC

Study	Immunocompromised patients								Immunocompetent patients							
	Asymptomatic	Cough	Expectoration	Fever	Chest pain	Dyspnea	Headache	Altered mental status	Asymptomatic	Cough	Expectoration	Fever	Chest pain	Dyspnea	Headache	Altered mental status
Yan Hu, et al	5	2	2	1	2	0	NA	NA	22	4	4	0	2	0	NA	NA
Dengfa Yang, et al	0	9	5	5	3	0	NA	NA	0	11	7	2	3	0	NA	NA
Junyan Qu, et al	3	37	NA	17	10	12	36	9	15	16	NA	3	5	4	3	1
Xin Sui, et al	2	8	8	14	3	1	10	6	5	9	9	2	2	2	2	2
Kaixiong Liu, et al	13	13	7	10	4	6	7	7	14	32	19	5	9	6	2	2
Lixuan Xie, et al	NA	NA	NA	NA	NA	NA	NA	NA	NA	NA	NA	NA	NA	NA	NA	NA
Jinquan Yu, et al	0	2	1	2	0	0	NA	NA	8	6	5	3	0	0	NA	NA
Kyoung Doo Song, et al	8	2	1	1	0	0	NA	NA	5	4	1	1	4	0	NA	NA
Jingqi Min, et al	2	15	10	3	4	NA	NA	NA	17	44	34	8	14	NA	NA	NA

### CT characteristics of immunocompetent and immunocompromised patients with PC

**Fig. 4:**
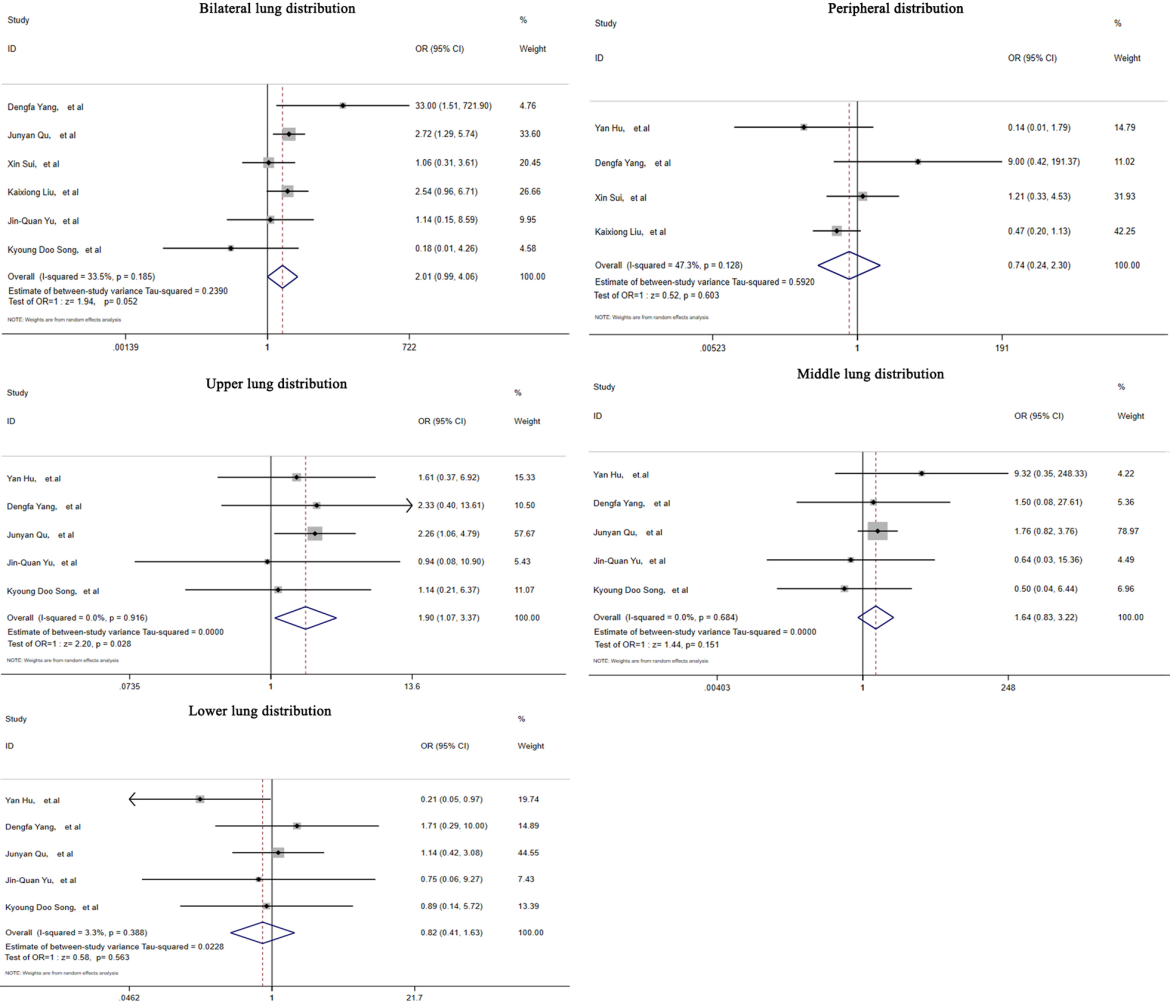
Forest plots depicting the comparisons of lesion distributions on thin-section CT in immunocompetent and immunocompromised PC patients

According to thin-section CT images, the bilateral lung lesion distribution was 50.0% in immunocompromised patients and 30.4% in immunocompetent patients. However, there was no significant difference in bilateral lung involvement between immunocompromised and immunocompetent PC patients (OR = 2.01, 95% CI (0.99–4.06), Z = 1.94, *p* = 0.052). Lesions were prominently distributed in the periphery in both immunocompromised and immunocompetent PC patients. In terms of the lesion distribution, the lower lobes seemed to contain more lesions (Table 3). A larger number of immunocompromised patients had lesion distribution in the upper lobes (OR = 1.90, 95% CI (1.07–3.37), Z = 2.2, *p* = 0.028) (Fig. 4).

**Table 3:** Lesion distributions in PC patients, according to thin-section chest CT

Lesion distributions	Immunocompromised patients				Immunocompetent patients			
	tN	Rate (%)	95% CI	I^2^ (%)	tN	Rate (%)	95% CI	I^2^ (%)
Bilateral lung distribution	178	50.0	36.2–64.7	60.4	157	30.4	18.6–42.1	57.1
Peripheral distribution	78	62.5	37.9–87.1	79.3	113	73.5	50.1–97.0	90.3
Upper lung involvement	129	53.8	34.5–73.2	67.1	115	40.8	28.2–53.4	48.6
Middle lung involvement	129	20.0	0.00–42.8	87.6	115	17.1	4.8–29.4	59.9
Lower lung involvement	129	71.8	55.5–88.0	57.7	115	78.6	70.6–86.5	11.6

‘tN’, total number of included cases; I^2^, heterogeneity test value

**Fig. 5:**
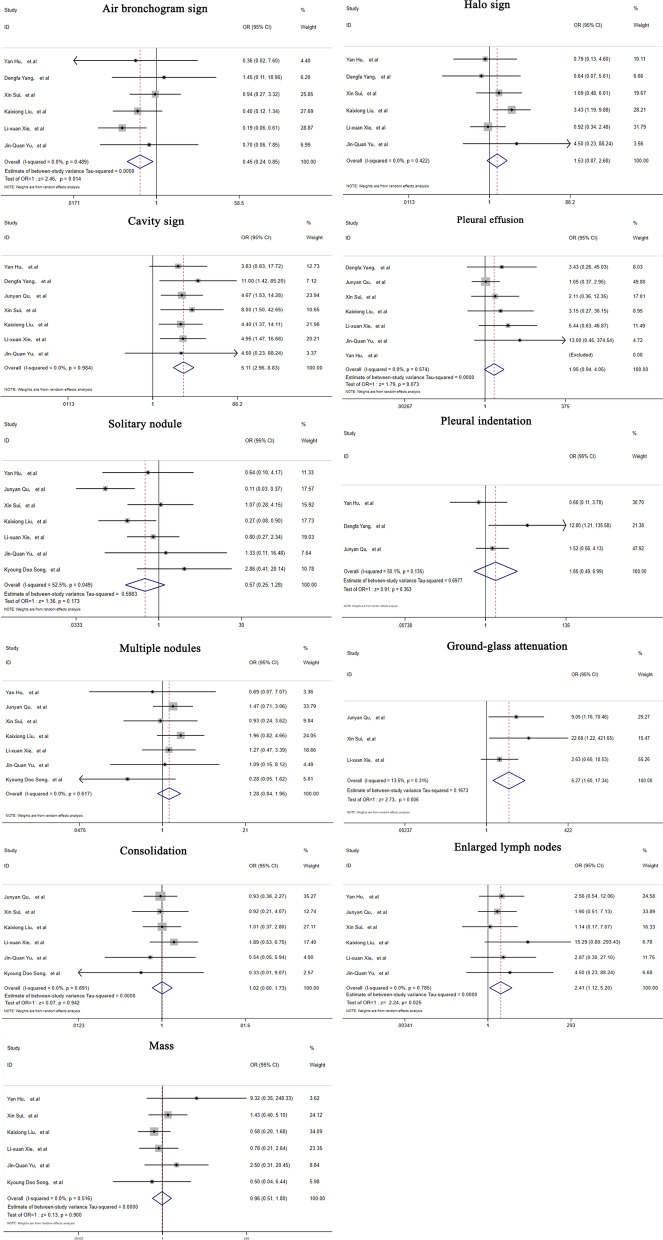
Forest plots depicting the comparisons of typical features on thin-section CT in immunocompetent and immunocompromised PC patients

The typical thin-section CT manifestations in PC patients were pulmonary nodules/masses, an air bronchogram sign, a halo sign, cavity sign, pleural effusion, ground-glass attenuation, consolidations, and enlarged mediastinal lymph nodes. The occurrences of these imaging findings are listed in Table 4 below. Generally, the proportions of patients with cavity sign (OR = 5.11, 95% CI (2.96–8.83), Z = 5.86, *p* = 0.00), ground-glass attenuation (OR = 5.27, 95% CI (1.60–17.35), Z = 2.73, *p* = 0.01), and mediastinal lymph node enlargement (OR = 2.41, 95% CI (1.12–5.20), Z = 2.24, *p* = 0.03) were significantly higher in the immunocompromised group than in the immunocompetent group (Fig. 5).

**Table 4:** Typical thin-section CT findings in PC patients

Typical CT manifestations	Immunocompromised patients				Immunocompetent patients			
	tN	Rate (%)	95% CI	I^2^ (%)	tN	Rate (%)	95% CI	I^2^ (%)
Air bronchogram sign	126	33.5	8.5–58.4	92.2	161	36.9	17.3–56.4	88.9
Halo sign	126	38.3	24.7–52.0	60.4	161	31.4	12.1–50.7	91.1
Cavitation	220	39.8	31.3–48.2	31.3	203	10.5	6.3–14.7	0.0
Pleural effusion	220	13.0	8.1–17.9	9.6	203	5.3	0.8–9.8	34.2
Ground-glass attenuation	161	23.2	13.9–32.5	41.8	89	4.7	0.0–11.8	40.8
Consolidations	212	21.3	15.7–27.0	0.0	173	19.2	13.4–25.0	0.0
Enlarged mediastinal lymph nodes	211	12.3	7.9–16.7	0.0	190	7.2	2.4–11.9	17.1
Solitary nodule	222	26.8	11.7–41.9	88.1	202	34.0	13.6–54.4	91.8
Multiple nodules	222	53.6	38.6–68.7	79.9	202	52.3	35.3–69.2	85.5
Mass	128	18.2	9.1–27.3	43.3	160	22.8	15.7–29.9	0.0

‘tN’, total number of included cases; I^2^, heterogeneity test value

## Discussion

Based on the detailed data extracted from 9 studies including 248 immunocompromised and 276 immunocompetent PC patients, our systematic review and meta-analysis provide a comprehensive description of clinical manifestations and CT findings in immunocompromised and immunocompetent PC patients.

The clinical manifestations in patients with PC are generally nonspecific, and patients can even be asymptomatic. Among the immunocompromised patients, asymptomatic infection occurred in up to 30.2%. Immunocompetent patients appeared to have a higher rate of asymptomatic infection (up to 40.8%). However, there was no significant difference in the proportion of asymptomatic cases between immunocompetent and immunocompromised groups. Some investigations have reported asymptomatic PC in more than 50% of patients [13, 25]. The reason for this difference might be differences in patient screening processes based on comorbidities or immune status. PC usually elicits several nonspecific respiratory symptoms, such as cough, expectoration, chest pain, and dyspnea. The incidence of these nonspecific respiratory symptoms was not significantly different between the immunocompetent and immunosuppressed PC patients. Nonspecific respiratory symptoms are likely to cause delays in PC diagnosis and subsequent proper treatments. Systemic syndromes with an overall low incidence, such as fever and headache, seemed to occur more frequently in immunosuppressed patients. The reason for more frequent systemic symptoms in immunocompromised patients might be due to the deficiency in immune surveillance in immunocompromised patients, resulting in failure to elicit a cryptococcus immune response. This immune surveillance deficiency results in more pulmonary exudative and necrotizing pathological changes, as well as the intrapulmonary or even systemic spread of Cryptococcus [26, 27]. In immunocompetent patients, cryptococcal infection tends to be localized due to phagocytosis by macrophages and granulomatosis formation. Therefore, immunocompetent patients tend to have mild pulmonary dissemination and fewer systemic symptoms.

According to thin-section chest CT images, nearly half of the immunocompromised PC patients had bilateral lung involvement. Even though less than one-third of the immunocompetent PC patients expressed bilateral lung involvement, the difference between the immunocompetent and immunocompromised patients was nonsignificant (*p* = 0.052). However, several retrospective studies revealed that immunocompromised PC patients are more likely to show bilateral lung lesions on chest CT than immunocompetent patients [18, 20]. Our meta-analysis failed to reveal a positive association, possibly because only 6 studies directly extracted data on unilateral or bilateral lung involvement, and the relatively small amount of data may cause bias. More than half of the total patient population showed a peripheral distribution of lesions. Our analysis indicated that PC tended to involve the lower lobes in both immunocompetent or immunocompromised patients, which is consistent with several retrospective studies [28]. Although upper lung involvement was not predominant in PC, it was relatively more common in immunocompromised patients.

The radiological features of PC, such as air bronchogram signs, halo signs, cavity, pleural effusion, ground-glass attenuation, consolidations, enlarged mediastinal lymph nodes, nodules and masses mimic other pulmonary infectious diseases and even malignant tumors [29]. This imaging finding similarity could be one of the reasons for the delay in PC diagnosis. Among these imaging findings detected by chest CT, cavitation, enlarged mediastinal lymph nodes, and ground-glass attenuation were more common in immunocompromised patients. Pleural effusion was rare in PC patients, especially in immunocompetent patients, with an incidence of only approximately 5%. Single or multiple nodules were the most commonly observed chest CT findings in both immunocompromised and immunocompetent PC patients according to our analysis and several other reports [20, 30, 31]. Similar to other pulmonary infectious diseases that induce the formation of granulomatous nodules during the disease process, the presence and architecture of granulomas, which present as “nodules” in chest CT imaging, are likely related to cryptococcus infection and intact host immune status. Consistent with other studies, our analysis concluded cavities sign within nodules, masses or other lesions occurred significantly more frequently in immunocompromised than in immunocompetent PC patients [21]. The difference may be caused by the inability to mount an effective immune response to localize cryptococcal infection in immunocompromised patients. The proliferating microorganisms destroy the adjacent lung tissue and promote the formation of cavities [26, 32]. Ground-glass attenuation was observed to be more common in immunocompromised patients. Several investigations of pulmonary fungal infections found that pulmonary exudation might be consistent with pulmonary hemorrhage caused by fungal infection. These pulmonary exudative lesions contain pathogenic microorganisms [32–34]. This imaging difference is also evidence of the inability to localize pulmonary cryptococcal infection in immunocompromised individuals. Mediastinal lymph node enlargement, which was more frequently encountered in immunocompromised individuals, is probably due to mediastinal lymphadenitis caused by the lymphatic spread of microorganisms in immunocompromised patients.

We acknowledge several limitations of our study. (1) A limited number of studies had available data related to the comparison of clinical and imaging features in immunocompromised and immunocompetent PC patients. Thus, for our analysis, we extracted only 9 suitable studies. (2) Due to the limited number of studies and available data, all patients included in our study were Asian. Ethnic homogeneity might have potential limitation to the results' generalizability. (3) In terms of the “immunocompromised” definition, the majority of studies defined “immunocompromised” status based on the presence of comorbidities or concomitant medications that caused immunosuppression. A few studies considered a combined evaluation of immune cell counts and immunoglobulin levels.

In conclusion, based on the limited available data, the immunocompromised PC group had a higher proportion of older adults (≥ 60 years) than the immunocompetent PC group. Among the nonspecific respiratory syndromes, we were unable to identify any symptoms that were significantly different between immunocompromised and immunocompetent PC patients. Nevertheless, several systemic symptoms, such as fever and headache, were more common in immunocompromised individuals. According to thin-section CT findings, lesions tended to be peripherally distributed in the lower lobes. However, upper lobe involvement was observed more frequently in immunocompromised patients. Similar as immunocompetent PC individuals, solitary or multiple nodules were the most common appearances in immunocompromised patients. Air bronchogram signs, halo signs, consolidations, and masses are possible radiologic features in immunocompetent and immunocompromised PC patients. Cavitation, ground-glass attenuation, and enlarged mediastinal lymph nodes are common radiologic features in immunocompromised PC patients but not immunocompetent individuals. By comparing the CT manifestations of immunocompetent and immunocompromised PC patients, we concluded the immune status results a significant impact on CT manifestations. For immunocompetent individuals with multiple nodules distributed in the lower lobes according to HRCT, PC should be taken into consideration [35]. Our results might help clinicians identifying the potential cryptococcal pneumonia and recognize the differences in clinical manifestations and CT findings in immunocompetent and immunocompromised PC patients.

## Supplementary Information

**Additional file 1.** Scores of the included studies.

## Data Availability

The data analyzed during this study are included in the published articles and its supplementary information.
