# Supplementary material for: Comparison of the clinical manifestations and chest CT findings of pulmonary cryptococcosis in immunocompetent and immunocompromised patients: a systematic review and meta-analysis
Source: BMC Pulm Med. 2022 Nov 11;22:415. doi: 10.1186/s12890-022-02175-9 (PMC9652893; doi:10.1186/s12890-022-02175-9)
Supplement: Supplementary file 1 — Additional file 1. Scores of the included studies. [file 12890_2022_2175_MOESM1_ESM.docx]

**Table. S1.** Risk of bias assessment

| Study | Selection | Comparability | Exposure | Total score |
| --- | --- | --- | --- | --- |
| Yan Hu, et al[9] | 3 | 1 | 2 | 6 |
| Dengfa Yang, et al[10] | 3 | 0 | 2 | 5 |
| Junyan Qu, et al[11] | 4 | 2 | 2 | 8 |
| Xin Sui, et al[12] | 3 | 1 | 1 | 5 |
| Kaixiong Liu, et al[13] | 3 | 1 | 2 | 6 |
| Li-xuan Xie，et al[14] | 3 | 0 | 2 | 5 |
| Jin-Quan Yu, et al[15] | 2 | 1 | 3 | 6 |
| Kyoung Doo Song, et al[16] | 3 | 2 | 2 | 7 |
| Jingqi Min, et al[17] | 3 | 2 | 2 | 7 |
